# Microgravity inhibits autophagy in human capillary endothelial cells in space flight

**DOI:** 10.1080/27694127.2022.2102363

**Published:** 2022-07-29

**Authors:** Ivana Barravecchia, Debora Angeloni

**Affiliations:** a Institute of Life Sciences, Scuola Superiore Sant’Anna, Via G. Moruzzi, 1 - 56124 Pisa, Italy; b Institute of Biorobotics, Scuola Superiore Sant’Anna, Via G. Moruzzi, 1 - 56124 Pisa, Italy

**Keywords:** Autophagy, capillary endothelial cells, chromosome territories, cytoskeleton, LC3B, microgravity, MTOR, RNA Sequencing, space radiation, telomere

## Abstract

Microgravity and space radiation (SR) are the two environmental factors that most affect human crews in space flight (SF). The endothelium is highly sensitive to gravitational unloading and several health problems reported by astronauts derive from endothelial dysfunction and impaired homeostasis. Recently, we found that space-flown, endothelial cells show cell softening, the presence of stress granules, reduced motility, profound cytoskeletal reorganization, an increased number of primary cilia, mitochondrial senescence, activation of DNA repair mechanisms, changes of chromosome territories, telomere shortening and increased apoptosis. The transcriptomic study showed activation of oxidative stress, inflammation and DNA damage repair pathways. In general, pathways for metabolism and a pro-proliferative phenotype are activated by microgravity and downregulated by SR. SR upregulates pathways for endothelial activation (hypoxia, cytokines, inflammation), DNA repair and apoptosis, promoting macroautophagy/autophagy flux and an ageing-like phenotype, which instead are downregulated by microgravity. Microgravity and SR exert opposite effects on the MTORC1 gene pathway: SR inhibits the pathway (with consequent enhancement of autophagy), while microgravity strongly stimulates MTORC1 (with consequent inhibition of autophagy). The sum of both contributions results in the net effect of autophagy inhibition in space-flown cells. Microgravity and SR should be considered separately to tailor effective countermeasures to protect astronauts’ health. Potentiation of autophagy is worthy of further investigation as a possible physiological countermeasure to SF-induced cell stress.

## Text

Mechanical cues regulate tissue structure and physiology. Properties and changes of the mechanical environment represent a challenge for cells, which must maintain a continuous homeostasis with their environment. Physical or chemical environmental shifts find the cells suddenly poorly suited to their surroundings, which they must rapidly adapt to. The lag between environmental changes and adjustment represents a period of severe disruption for the cell, which activates stress-management and adaptation pathways to regain homeostasis. The induction of autophagy is one such response to mechanical changes. A few recent works associate altered autophagic response to microgravity, both in ground simulations and space flight (SF).

Recently, we described the response of human microvascular endothelial cells (HMEC-1) to SF [1], focusing on this cell type because the endothelium exerts a fundamental role in maintaining the homeostasis. Many health issues reported by astronauts are linked to endothelial suffering, shared to a certain degree by the elderly, severely sedentary individuals, and healthy volunteers in bed-rest studies.

In space-flown cells, we observed a range of phenotypic morphological changes that were significantly corroborated by a transcriptomic study. The adaptive response to SF exerted by living beings, from single cells up to whole organisms, is brought about by several environmental factors, including microgravity and space radiation (SR) among the most peculiar.

Our study, thanks to adequate sample numbers and proper reference samples set on ground and at 1*g* in the onboard centrifuge, allowed us to distinguish the respective contribution of microgravity and SR to the combined response to SF.

In general, pathways for metabolism and a pro-proliferative phenotype are activated by microgravity and downregulated by SR. SR turns on pathways for endothelial activation (hypoxia, cytokines, inflammation), DNA repair and apoptosis, promoting autophagy flux and an ageing-like phenotype; the same are downregulated by microgravity.

**Figure1.**
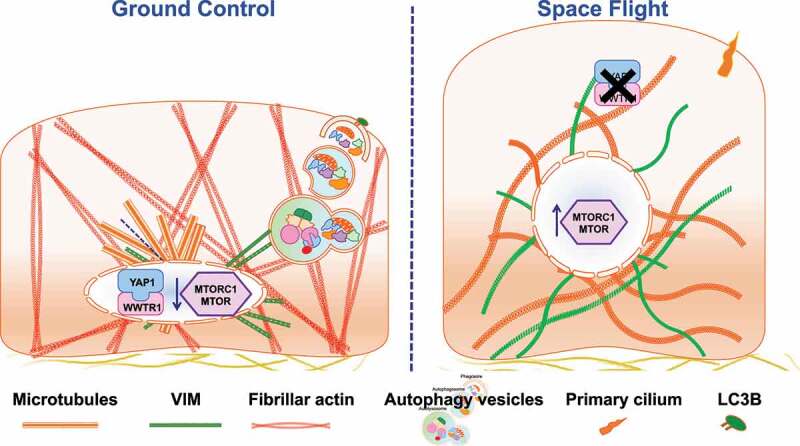
Release of autophagic vesicles requires cytoskeleton integrity and YAP1 nucleo-cytoplasm shuttling. In space flight, YAP1 distribution is undetectable and the adaptive response to gravitational unloading causes a profound rearrangement of cytoskeleton compartments, with loss of actin stress fibers and compensatory distribution of microtubules and intermediate filaments; absence of the LC3B marker (seen with immunofluorescence) and the upregulation of MTORC1 and PI3K-AKT-MTOR signaling pathways (seen with RNA-Seq) suggest the inhibition of autophagic flux.

Interestingly, immunofluorescence showed that the autophagic marker MAP1LC3B/LC3B (microtubule associated protein 1 light chain 3 beta) was almost undetectable in SF [1], suggesting inhibition of the autophagy flux. This hypothesis was supported by transcriptomic data showing activation, in SF, of the MTORC1 and PI3K-AKT-MTOR signaling pathways, key negative controllers of autophagy. Remarkably, bioinformatics analysis of the transcriptome of space-flown and reference cells, showed that microgravity and SR act on the MTORC1 gene pathway with opposite effects: SR inhibits the pathway (with consequent enhancement of autophagy), whereas microgravity strongly stimulates MTORC1 (with consequent inhibition of autophagy). The sum of both contributions results in the net effect of autophagy inhibition in space-flown cells, suggesting autophagy impairment might contribute to the well-known frailty of ECs in SF. Autophagy requires an efficient YAP1 nucleus-cytoplasm shuttling mechanism, which in turn requires cytoskeleton integrity. Space-flown samples show marked cytoskeletal disruption and loss of YAP1 signaling, in addition to activation of stress-related pathways [1] (Figure 1).

This means that stimuli that on Earth would increase autophagy, in space are insufficient to activate the autophagic flux. This also suggests that inducing autophagy in space could protect ECs from the negative effects of microgravity, if not with restoring cytoskeletal integrity at least by counteracting downstream toxic effects through the elimination/recycling of damaged constituents.

Recently, space biomedical research has been interested in the topic of autophagy, reporting different results in a plethora of models. Out of 30 papers currently in PubMed concerning autophagy and microgravity, only five studied ECs. All but ours [1], were performed with microgravity simulators, and used macrovascular models of ECs (HUVEC). Given the scarce opportunities of access to space, simulation experiments are important; however, they cannot replace the experiment in SF, due to the extreme complexity of the entailed conditions. Noteworthy, our study is the first to observe a model of microvascular ECs and the first realized in SF, and it reports inhibition of autophagy due to microgravity in SF, that is the opposite of what is observed in all ground simulations of microgravity with macrovascular ECs.

Regardless of the experimental cell model, weightlessness is known to induce substantial changes of cardiovascular function, and the peculiar effect of heavy ions in cosmic rays might exacerbate these changes substantially. Alteration of the cardiovascular function in-flight causes physiological risks also in the post-flight period. Therefore, our report of inhibition of autophagic flux in capillary ECs in real SF, the first of its kind to our knowledge, is important because it suggests the need to explore pharmacological treatments for preserving the endothelial and the entire cardiovascular function.
